# Dispersal capacity explains the evolution of lifespan variability

**DOI:** 10.1002/ece3.4073

**Published:** 2018-04-19

**Authors:** Ismael Galván, Anders P. Møller

**Affiliations:** ^1^ Departamento de Ecología Evolutiva Estación Biológica de Doñana CSIC Sevilla Spain; ^2^ Ecologie Systématique Evolution Université Paris‐Sud, CNRS, AgroParisTech, Université Paris‐Saclay Orsay Cedex France

**Keywords:** aging, birds, comparative studies, dispersal, evolution of lifespan

## Abstract

The evolutionary explanation for lifespan variation is still based on the antagonistic pleiotropy hypothesis, which has been challenged by several studies. Alternative models assume the existence of genes that favor aging and group benefits at the expense of reductions in individual lifespans. Here we propose a new model without making such assumptions. It considers that limited dispersal can generate, through reduced gene flow, spatial segregation of individual organisms according to lifespan. Individuals from subpopulations with shorter lifespan could thus resist collapse in a growing population better than individuals from subpopulations with longer lifespan, hence reducing lifespan variability within species. As species that disperse less may form more homogeneous subpopulations regarding lifespan, this may lead to a greater capacity to maximize lifespan that generates viable subpopulations, therefore creating negative associations between dispersal capacity and lifespan across species. We tested our model with individual‐based simulations and a comparative study using empirical data of maximum lifespan and natal dispersal distance in 26 species of birds, controlling for the effects of genetic variability, body size, and phylogeny. Simulations resulted in maximum lifespans arising from lowest dispersal probabilities, and comparative analyses resulted in a negative association between lifespan and natal dispersal distance, thus consistent with our model. Our findings therefore suggest that the evolution of lifespan variability is the result of the ecological process of dispersal.

## 1 INTRODUCTION

With only a few exceptions, organisms deteriorate as they age and consequently die, but large variation in longevity still exists among species (Finch, 1990). This phenomenon can only be fully understood from a comprehensive view of both proximate mechanisms and ultimate functions. From the perspective of the proximate mechanisms, advances made in the last two decades have permitted to establish that certain cellular characteristics determining the rate of generation of reactive oxygen species (ROS) are responsible for the aging process (Pamplona & Barja, 2007, 2011). The most comprehensive comparative study of these factors to date regarding the number of species (it used 107 species of birds) has found that fatty acid characteristics of cellular membranes have a prominent causative role in the aging process, species with longer maximum lifespans being those with higher proportions of long and monounsaturated fatty acids in their membranes (Galván et al., 2015).

The question then arises as to why high proportions of long and monounsaturated fatty acids have not evolved in all species, given that this would maximize their lifespan and should therefore be promoted by natural selection, as aging is clearly detrimental for the fitness of individual organisms. In other words, why has a variability of lifespans evolved among species, and particularly, why the cellular characteristics promoting ROS production and aging (i.e., high proportion of short and polyunsaturated membrane fatty acids) and the corresponding short lifespans have evolved in some species? This question was already formulated by Darwin (1859), but in contrast to the proximate mechanisms of aging, the study of longevity from the perspective of the ultimate functions, that is, the evolution of aging and lifespan, is still based on Williams’ (1957) antagonistic pleiotropy hypothesis: high mortality rates promote rapid reproduction, and direct selection for rapid reproduction leads to indirect selection for shorter lifespan. The evolutionary theory of aging has thus remained almost intact for more than half a century, assuming that high senescence rates and short‐lifespan result from an increase in environmentally mediated mortality (Austad, 1993). While this hypothesis has sometimes been supported by data from wild populations of animals (Chen & Maklakov, 2012; Valcu, Dale, Griesser, Nakagawa, & Kempenaers, 2014), other empirical studies have frequently called into question the claimed role of extrinsic mortality in promoting senescence and the evolution of short lifespan (Garratt et al., 2015; Hämäläinen et al., 2014; Reznick, Bryant, Roff, Ghalambor, & Ghalambor, 2004; Ricklefs, 2008; Williams, Day, Fletcher, & Rowe, 2006).

Maybe as a response to this incapacity of the antagonistic pleiotropy hypothesis to provide a general explanation for the evolution of lifespan variability, some theoretical models have appeared in the last years based on the idea of programmed aging, stating that organisms have a genetically fixed senescence rate that is favored by natural selection because senescence may be adaptive in certain circumstances (Longo, Mitteldorf, & Skulachev, 2005; Mitteldorf, 2016; Mitteldorf & Martins, 2014; Mitteldorf & Pepper, 2009; Werfel, Ingber, & Bar‐Yam, 2015). These models have the important limitation of dealing with group selection, as they assume that senescence benefits lineages by avoiding overpopulation and associated problems such as resource depletion and epidemics, and thus lack an evolutionary logic (Kowald & Kirkwood, 2016). In fact, no genes exist that promote aging (de Grey, 2015).

**Figure 1:**
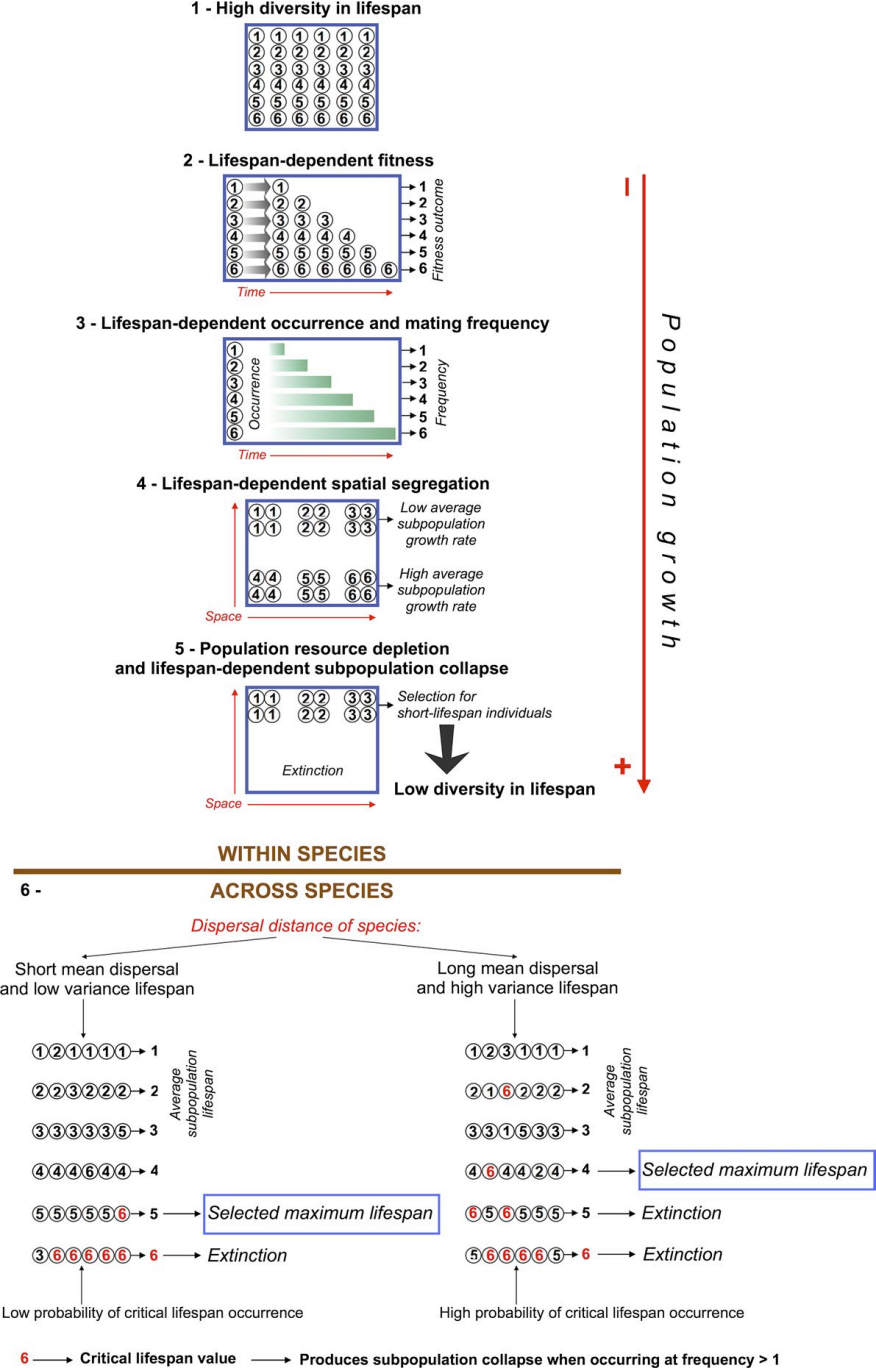
Schematic representation of the evolutionary model for lifespan, with hypothetical lifespan values ranging from 1 to 6. Circles represent individual organisms with a given composition of genes regulating lipid composition that confers the lifespan indicated by the inside numbers. Steps 1–5 occur within species, only if dispersal is sufficiently low. Step 6 represents the interspecific process that may lead to a better maximization of viable lifespan values in species that disperse short distances on average. Numerical values shown in the figure are examples for illustrative purposes only

Here we propose that the evolution of lifespan is based on the ecological process of dispersal and does not depend on extrinsic mortality nor assume any adaptive benefit of aging, thus avoiding the above‐mentioned limitations of group selection. Dispersal has previously been proposed in a theoretical model as a determinant of aging assuming that shorter dispersal distances create more competition for resources and shorter lifespans are then favored under such conditions because it would be beneficial for the lineages (Dytham & Travis, 2006), therefore carrying the problems of group selection and programmed aging. Here we first provide theoretical arguments by which a similar dependence of lifespan evolution on dispersal distance can be achieved with basic concepts of population dynamics without the need of assuming adaptive group benefits or a genetic aging clock. Our proposal can be summarized in the following steps (Figure 1):

1. There are no genes that hasten aging (de Grey, 2015), but there are genes that regulate lipid composition which in mammals shows evidence of selection (Jobson, Nabholz, & Galtier, 2010). Different factors affect lifespan such as the content of methionine in proteins (Aledo, Li, de Magalhães, Ruiz‐Camacho, & Pérez‐Claros, 2011; Pamplona & Barja, 2007), but as mentioned above fatty acid characteristics seem to constitute one of the main proximal mechanisms determining lifespan (Galván et al., 2015). Thus, it can be assumed that the starting state is a population composed of all individual organisms in a given species in which every individual has a certain conformation regarding the genes that regulate lipid composition, so that all possible values of gene forms or expressions are represented in the population. As a consequence, all possible values of lifespan are represented in the population, causing lifespan not to be common in all individuals of a species in this initial stage, but variability in lifespans exists instead. In fact, in all comparative studies of lifespan a single lifespan value is assigned to each species, typically the maximum lifespan, but intraspecific variation in lifespan can be very large (Munch & Salinas, 2009). Our model assumes a progressive intraspecific reduction of this variation in lifespan (see below).
2. Assuming that all individuals in the population have similar breeding success and therefore similar fecundity, the population starts growing, and growth remains constant during the entire process. In this process, individuals with the genetic conformation that provide certain membrane fatty acid characteristics leading to longer lifespans (Galván et al., 2015) achieve greater fitness benefits than individuals with the genetics of fatty acid composition that lead to shorter lifespans.
3. Assuming that individuals maintain an active reproductive capacity throughout their lives and re‐mate following the death of their mate, and given the heterogeneity in lifespans, individuals with longer lifespans coexist during longer periods of time. Even if random mating occurs, this will make mating events to occur more frequently between individuals with longer lifespans than between individuals with shorter lifespans. This may constitute the germline to spatial segregation by lifespan, so that individuals with shorter lifespans would tend to aggregate in spatial subpopulations. The main force leading to this spatial segregation, however, would be dispersal (see next step).
4. Natal dispersal, defined as the movement of organisms between birth and breeding sites, can avoid, if long enough, the fixation of deleterious mutations and the problems derived from inbreeding by favoring gene flow (Keller & Waller, 2002). Thus, if short enough, natal dispersal can fix certain genetic characteristics around a source site in a subpopulation. Indeed, dispersal plays a key role in determining the structure of wild populations (Bohonak, 1999). Therefore, we assume that all individuals in a population have a similar dispersal capacity, and that the average natal dispersal is sufficiently short to limit gene flow to ensure that more interactions occur between individuals within the subpopulation than between individuals within and outside a subpopulation. The forms or expression of genes coding for lipid composition that lead to short lifespans would tend to accumulate around individuals that carry those genetic characteristics. This will avoid the disappearance of spatial subpopulations arisen in the previous stage. As a consequence, subpopulations will be created where lifespan is, on average, shorter, and growth rate is lower than the average of the metapopulation.
5. Frequently, extrinsic mortality is insufficient to limit growth in the wild, and collapses are often observed in natural populations (Le Galliard, Fitze, Ferrière, & Clobert, 2005; Shennan et al., 2013). Assuming that the availability of essential resources is finite, and that they are uniformly distributed, population collapse is less likely to occur in subpopulations with short lifespan than in the rest of the population due to their reduced growth rate. It must be reiterated here that breeding rate is assumed to be the same for all individuals regardless of lifespan (see point 2 above), and this is why individuals with shorter lifespan should deplete resources at a lower rate than individuals with longer lifespan. Assuming that population collapse will always occur at some time as growth increases, it will occur first in the parts of the populations that are segregated from the short‐lifespan subpopulations, leading to population extinction. Subpopulations with the lowest lifespan values survive and thus the genetic characteristics coding for the fatty acid composition that lead to such low lifespans evolve. Of course, this should not always happen, as the population could stop growing or achieve a stationary equilibrium of size. Our model is thus dependent on a continuous population growth, so the reduction in lifespan variation within species would be only observed when this occurs.
6. The previous step should occur within every species. The final result, however, may differ between species depending on their average dispersal capacity, which will determine the heterogeneity in lifespan values within the short‐lifespan subpopulations. This is because, if natal dispersal is long, the probability that a dispersing individual interacts with others with different lifespans is high, so the short‐lifespan subpopulations will contain individuals with a high variability in lifespan values. By contrast, if natal dispersal is short, the short‐lifespan subpopulations will contain more individuals with similar lifespan. Even if gene flow is sufficiently limited to allow the spatial segregation of short‐lifespan (on average) subpopulations, these will be more heterogeneous regarding individual lifespan in species with average longer dispersal distances than in species with shorter dispersal distances. This means that species with shorter dispersal distances show the potential to have a homogeneous short‐lifespan subpopulation (i.e., with all individuals having the same lifespan) for every possible value of lifespan. Assuming that in all species a critical value for lifespan exists over which, for their particular breeding and mortality rates, the population collapses, this in turn means that species with shorter dispersal distance will be able to maximize the lifespan value that is viable before reaching the collapsing value. In species with longer dispersal distance, by contrast, the short‐lifespan subpopulations that contain the critical collapsing value of lifespan will be more heterogeneous, ensuring that the collapsing value will coexist with lower lifespan values in some short‐lifespan subpopulations. These subpopulations will collapse because they contain the collapsing value, leading to the disappearance of individuals with shorter lifespans that coexist in the subpopulations.

As a consequence, greater dispersal capacity should promote the evolution of shorter lifespans, and variability in dispersal capacity should be associated with lifespan variability among species. Here we test this model with individual‐based simulations and with empirical data from wild populations of 26 species of birds.

## 2 METHODS

### 2.1 Individual‐based simulations

Individual‐based simulations followed the island model of dispersal and was related to a model described elsewhere (Aguilée, Shaw, Rousset, Shaw, & Ronce, 2013), with modifications to achieve the specifications of the verbal model provided above. The effect of dispersal capacity was simulated here by considering dispersal probability, that is, the probability that an individual changes subpopulation at the dispersal stage (Aguilée et al., 2013). The following parameters thus comprised the model: number of populations (*npop*), number of individuals in each population (*nindiv*), critical frequency of individuals with the critical lifespan leading to population collapse (*critfreq*), maximum possible lifespan (*maxlifespans*), dispersal probability (*pdisp*), and maximum number of generations (*tmax*). Additionally, we included the effect of mutation during simulations. For simplicity, we considered a mode of clonal reproduction with mutation, assuming a mutation rate of 10^−9^ per genome. We allowed lifespan to evolve for 1,000 generations, after which we calculated the mean lifespan assigned by the simulations to the metapopulation under different *critfreq* values. Simulations were run in R version 3.3.1 (R Core Team, 2017). Details of the simulations are given in the R code provided in Appendix S1.

### 2.2 Bird lifespans

We obtained information on maximum longevity of all species from the European bird ringing organization EURING (http://www.euring.org). Longevity records provide reliable information on maximum lifespan if records are adjusted for sampling effort (Møller, 2006). Among 130 species of common birds in Europe with longevity records, the total number of recoveries and recaptures of banded birds in Europe ranged from 118 to 187,764 (http://www.euring.org), in total 2,313,199. Therefore, we used the total number of recoveries reported by EURING as a measure of sampling effort for all records of longevity. Maximum lifespan values were thus adjusted for sampling effort (Appendix S2).

### 2.3 Natal dispersal distance

We used geometric mean natal dispersal distance as reported by Paradis, Baillie, Sutherland, and Gregory (1998). We assume that these estimates obtained from large numbers of ringed birds in United Kingdom also extend to Europe in general (see also Belliure, Sorci, Møller, & Clobert, 2000).

### 2.4 Genetic variability

Our evolutionary model for lifespan assumes that dispersal capacity drives the spatial distribution of the genetic characterization of lifespan, which is determined by the genes that regulate lipid composition. However, the geographic genetic variability of natural populations is large among species, and all them are subject to genetic homogeneity produced by gene flow (Slatkin, 1987). Thus, the potential of dispersal capacity to exert an effect on lifespan should be higher in species with a low prevalent overall genetic variability in its populations than in species with a high genetic variability, as the formation of homogeneous genetic groups should be more difficult in the latter. Therefore, we controlled for the overall genetic variability of species when testing for an association between lifespan and natal dispersal distance. Genetic variability was estimated as the observed heterozygosity (H_0_) in the species’ populations (Wright, 1922).

We obtained information on genetic variability for microsatellites in birds by a search of Web of Science using the terms microsat*, micro‐sat*, and bird*. In total, we obtained 7,996 entries for 380 species with information on H_0_ (Appendix S2).

### 2.5 Body size

We considered the mean body mass of species as a surrogate of body size and used it as a predictor in the analyses to avoid detecting effects of natal dispersal distance on maximum lifespan that may arise because of associations between body mass and maximum lifespan (Valencak & Azzu, 2014).

Body mass was recorded as the mean mass of males and females from the breeding season, as reported by Cramp and Perrins (1977). If more than one estimate was reported by that source, we used that with the largest sample size (Appendix S2).

### 2.6 Statistical analyses

Bird species are evolutionarily related through their common phylogenetic history, which can lead to overestimation of degrees of freedom if phylogenetic relationships are not taken into account. Thus, we obtained a phylogeny on which we modeled the relationship between lifespan and the response variables described above for the 26 species included in our study. Our original sample was reduced to 26 species due to missing information for some species. For this, we obtained 1,000 probable phylogenies with branch lengths expressed as proportions of nucleotide substitutions available in http://www.birdtree.org (Jetz, Thomas, Joy, Hartmann, & Mooers, 2012) and then calculated the least‐squares consensus phylogenetic tree from the mean patristic distance matrix of the set of 1,000 phylogenies using the package *phytools* (Revell, 2012) in R version 3.3.1 (Appendix S3).

After obtaining the phylogenetic relationships among the 26 species in the study, we used a phylogenetic generalized least‐squares (PGLS) model to control for phylogenetic effects in the analysis of the relationship between lifespan and natal dispersal distance, genetic variability, and body size. We ran PGLS models in R with an unpublished function by R. Freckleton (Univ. Sheffield) (pglm3.4.r, available on request), and using the appropriate packages (*ape*,* MASS* and *mvtnorm*). This function performs PGLS and calculates the measure of phylogenetic correlation (λ) established by Pagel (1999). λ is a likelihood ratio test that assumes a constant‐variance random effects model of evolution (i.e., Brownian model) and adjusts the analyses for the degree of dependence among traits, considering that this degree can vary between a complete independence of data on phylogeny (λ = 0) and a complete dependence of data on phylogeny (λ = 1). For our model, the maximum‐likelihood (optimal) value of λ given the phylogenetic hypothesis did not differ from 0 (χ = −8.36 × 10^−4^, *p* = 1), thus indicating phylogenetic independence.

We therefore tested our model without considering phylogenetic effects, analyzing the relationship between the response variable (maximum lifespan) and natal dispersal distance, H_0_ and body mass by means of a general linear model (GLM). We weighted the number of recoveries per species to control for sampling effort in the calculation of maximum lifespan, as maximum lifespan obviously increases with sampling effort (Møller, 2006, 2007). Weighting was made computing moment statistics based on the sum of the weight value for the weighting variable (i.e., number of recoveries per species) and degrees of freedom as the sum of the weighted values minus one. All variables were log_10_ transformed to ensure normality assumptions, except H_0_, which was arcsine square‐root transformed. Both raw regression coefficients (*b*) and standardized regression coefficients (β) estimated from the model are provided.

## 3 RESULTS

### 3.1 Individual‐based simulations

**Figure 2:**
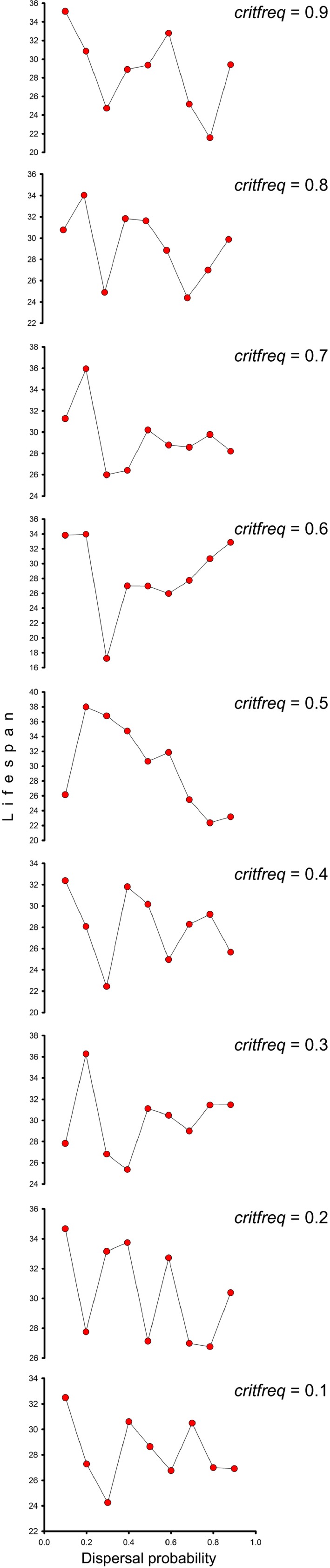
Simulated mean population lifespan variation with dispersal probability under different frequencies of individuals with the critical lifespan that leads to population collapse (*critfreq*). Results for lifespan evolution after 1,000 generations, mutation rate of 10^−9^ per genome, and the following values for model parameters are shown: *npop* = 50, *nindiv* = 100, *maxlifespans* = 50

After allowing lifespan to evolve after 1,000 generations under different critical frequencies of individuals with the critical value of lifespan leading to population collapse, the maximum values of the resulting mean lifespan of the metapopulation were obtained for the two lowest values of dispersal probability considered (i.e., 0.1 and 0.2) (Figure 2). This suggests that dispersal constrains lifespan evolution.

### 3.2 Comparative study of birds

**Figure 3:**
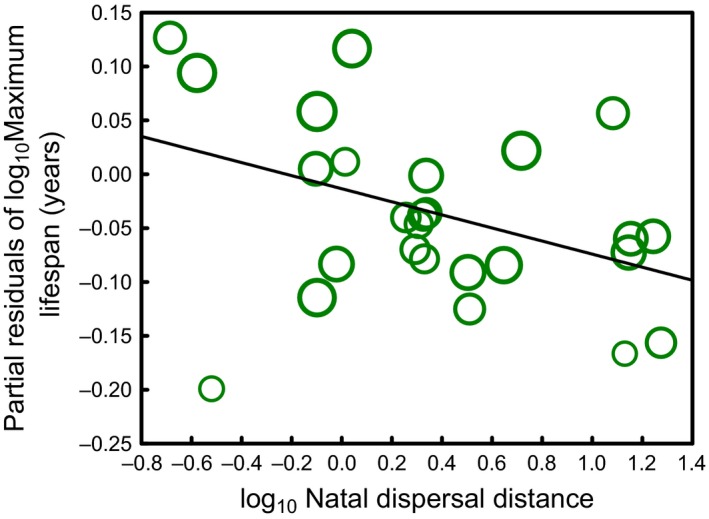
Relationship between maximum lifespan and natal dispersal distance in 26 species of birds. The residual figures of the response variable (i.e., partial effects after applying a general linear model without natal dispersal distance) are shown. Data points increase logarithmically in size with the number of recoveries used to estimate maximum lifespan. The line is the best fit line

The GLM explained a large proportion of variance (72.0%) in maximum lifespan among species (*F*_3,22_ = 18.89, *p* < .0001). As predicted by our evolutionary model, natal dispersal distance had a significant negative effect on maximum lifespan (*b* = −0.09, β = −0.37, *F*_1,22_ = 8.79, *p* = .007) after controlling for genetic variability (*b* = −0.17, β = −0.12, *F*_1,22_ = 0.77, *p* = .388) and body mass (*b* = 0.17, β = 0.78, *F*_1,22_ = 42.22, *p* < .0001) (Figure 3).

## 4 DISCUSSION

A large body of empirical research demonstrates that senescence is not the result of natural selection favoring short lifespans because of any adaptive benefits, but the result of certain metabolic processes that are essential for life to occur but also produce cytotoxic ROS and subsequently cell death and phenotype deterioration (Schaar et al., 2015). The most comprehensive study to date on proximal mechanisms determining lifespan variation between species (107 species of birds) suggests that the composition of cell membrane fatty acids is the best determinant of cellular ROS production relevant for aging, according to the homeoviscous longevity adaptation hypothesis (Galván et al., 2015). It then raises the question of why natural selection has not always favored the evolution of fatty acid characteristics that allow achieving long lifespans, if fitness for a given breeding rate increases with survival. Only the existence of a constraint could avoid a prevalent evolution of long lifespans. Our arguments, which are supported by individual‐based simulations and empirical data from 26 species of birds, state that the dispersal capacity of organisms constitutes such a constraint: short lifespans have evolved in species that disperse longer distances because long dispersal movements may impair the maximization of viable lifespans regarding population growth. This study thus suggests that the evolution of lifespan variability is the result of an ecological process. A previous study actually showed that ecological interactions between birds and their predators explain interspecific differences in rates of survival and senescence (Møller, 2010), which is related to the role of ecology in determining lifespan variability that the present study suggests. Our model assumes that this evolution is dependent on species’ populations that continuously grow and eventually collapse and where dispersal is sufficiently low to limit gene flow and allow the formation of spatially segregated subpopulations with similar average individual lifespan (see Introduction). Therefore, lifespan variability would only evolve when these conditions occur, which may be useful for future empirical demonstrations of the model in extant species.

Dispersal was previously proposed as an ecological process driving lifespan evolution by Dytham and Travis (2006). These authors concluded that longer lifespans have evolved in species with longer dispersal distances, just the opposite to the predictions of our model and the result of our comparative analysis of empirical data of birds. However, Dytham and Travis’ (2006) theoretical model considered that short lifespans may evolve when dispersal distances are short because of the benefits that reducing resource competition may confer to the lineages, thus basing their arguments on group selection. The underlying assumption of models for the evolution of lifespan that depends on hypothetical benefits that groups (i.e., species) would obtain at the expense of individual lifespan reduction is that some genes favor aging, which is a wrong assumption as it is not supported for any empirical study (de Grey, 2015). In fact, no evolutionary model for lifespan based on group selection benefits has been supported by empirical data.

It must be noted that our model considers the differential extinction of subpopulations on the basis of the average lifespan of their individual components, while group selection would assume that selection minimizes the extinction rate of subpopulations (Wynne‐Edwards, 1986). Thus, our model does not consider selection acting on group characteristics, but on individual characteristics (individual lifespan) that become grouped because of spatial segregation. Therefore, our model provides arguments for how dispersal could be the causative agent for the evolution of lifespan variability without assuming any group benefits, only the potential of short dispersal distances to produce the spatial segregation of individuals of similar lifespan and the potential of segregated individuals with shorter lifespans to resist more time to resource depletions. Thus, short‐lifespan individuals would not be selected because of any benefits for their groups, but would just be the remaining population components after a hypothetical extinction of part of the metapopulation because of resource depletion. We assume that every individual has a certain characterization (i.e., forms or expression levels) of the genes that regulate membrane fatty acid composition, which in turn determines their lifespan (Galván et al., 2015). Therefore, we consider that natural selection acts, through the ecological process of dispersal, on this variation in the genetic characterization of fatty acid composition genes and the corresponding lifespan values rather than the existence of genes favoring aging. Indeed, a basis of our model is the dispersal‐dependent reduction of lifespan variability that takes place within species from a hypothetical initial state in which individuals with all possible lifespan values coexist (Figure 1). Evidence of selection in the genes that regulate lipid composition in mammals (Jobson et al., 2010) may be in accordance with our model, as it suggests that the genes potentially determining lifespan have experienced a process of reduction in variability like the one we are proposing here. The fact that the prediction of our model was supported by both individual‐based simulations and empirical data on birds suggests that the model is correct and that group selection, which predicts a positive association between lifespan and dispersal distance (Dytham & Travis, 2006), is not at work during the evolution of aging and lifespan. It may represent another example of the incapacity of nondarwinian arguments such as group selection to provide realistic predictions in evolutionary biology (Parker & Maynard Smith, 1990).

The results of our comparative study of 26 species of birds showed congruence with the proposed eco‐evolutionary scenario, indicating that species with longer lifespans are those that disperse less before establishing at the breeding grounds. This negative association between lifespan and natal dispersal distance across species agrees with the theoretical mechanism by which spatially segregated subpopulations should be more homogeneous regarding the lifespan of their individual components in species with shorter than in species with longer dispersal distances because of limited gene flow in the former (Keller & Waller, 2002). Assuming that subpopulations experience resource depletion and collapse, which is often observed in the wild despite extrinsic mortality (Le Galliard et al., 2005; Shennan et al., 2013), every species should have, given its characteristic population growth rate, a critical value for lifespan that would generate resource depletion and collapse when it appears at a frequency that is above a certain threshold among the individual components of the subpopulations (*critfreq* in our individual‐based simulations). The difference in the homogeneity in average lifespan in spatially segregated subpopulations between species with short and long dispersal distances should therefore ensure that the probability that the collapsing lifespan value appears at a frequency above the critical threshold value is higher in long dispersal than in short dispersal species. This should in turn make that the probability that a subpopulation with an average lifespan near the upper critical lifespan value becoming viable is higher in species with shorter dispersal distances than in species with longer dispersal distances, thus explaining the higher likelihood of finding long maximum lifespans among species that disperse less. This theoretical consequence was supported by the comparative study of birds even after controlling for confounding factors. First, we controlled our comparative analyses for the genetic population variability of the species, suggesting that species that disperse longer have a reduced capacity to form homogeneous subpopulations regarding lifespan because of the constraining effects of dispersal and not because these species had a higher general genetic variability. Furthermore, body size is known to be a general predictor of maximum lifespan across species (Valencak & Azzu, 2014), and by controlling for this factor, our results thus suggest that lifespan co‐evolves with natal dispersal distance because of the effects of dispersal and not because of potential associations between dispersal distance and body size.

Interestingly, the comparative study on the ecological factors affecting lifespan variation that has used the highest number of species to date concluded that the capacity to fly was the most important determinant of lifespan in birds and mammals (Healy et al., 2014). These authors interpreted that the effect of flight capacity on lifespan variation might be due to the reduction in extrinsic mortality exerted by flight as predicted by Williams’ (1957) antagonistic pleiotropy hypothesis, but given the lack of general validity of this hypothesis (see Introduction), and the overlooked effect of dispersal capacity in the evolutionary studies of lifespan, the possibility that common lifespans in flying species is due to potentially common natal dispersal capacity instead of extrinsic mortality reduction should now be investigated.

In conclusion, we provide the first evolutionary model for lifespan alternative to the antagonistic pleiotropy hypothesis that is not based on genetic programming of aging and group selection benefits and is supported by empirical data from natural populations. The antagonistic pleiotropy hypothesis has received mixed support from empirical studies (e.g., Reznick et al., 2004), suggesting that the role of extrinsic mortality in determining lifespan variability may not apply to all organisms. Our model proposes that the evolution of lifespan variability is the result of constraints imposed by the dispersal capacity of species.

## CONFLICT OF INTEREST

None declared.

## AUTHOR CONTRIBUTIONS

I.G. conceived the idea and the verbal model, conducted the analyses of data, and wrote the manuscript. A.P.M. collected the data on bird lifespans, natal dispersal distance, genetic variability, and body size.
