# Supplementary material for: Dispersal capacity explains the evolution of lifespan variability
Source: Ecol Evol. 2018 Apr 19;8(10):4949–57. doi: 10.1002/ece3.4073 (PMC5980329; doi:10.1002/ece3.4073)
Supplement: Supplementary file 2 [file ECE3-8-4949-s002.docx]

**Appendix S2.** Maximum lifespan, sampling effort, natal dispersal distance, population genetic variability (observed heterozygosity, H_0_) and body mass for the 26 species of birds included in the study.

| **Species** | **Maximum lifespan (years)** | **Sampling effort**  **(no. recoveries)** | **Natal dispersal distance**  **(geometric mean, km)** | **H_0_** | **Body mass (g)** |
| --- | --- | --- | --- | --- | --- |
| *Accipiter gentilis* | 18.7 | 10610 | 18.84 | 0.559 | 1139.5 |
| *Acrocephalus scirpaceus* | 12.9 | 98244 | 5.215 | 0.63 | 11.8 |
| *Aegithalos caudatus* | 10.7 | 10815 | 2.176 | 0.636 | 8.8 |
| *Alauda arvensis* | 9.4 | 2276 | 0.302 | 0.61 | 36.4 |
| *Buteo buteo* | 28.7 | 17142 | 12.1 | 0.539 | 806.5 |
| *Carduelis flammea* | 10.7 | 7100 | 2.139 | 0.765 | 13.05 |
| *Corvus corone* | 17.9 | 12359 | 3.24 | 0.631 | 544.5 |
| *Corvus frugilegus* | 20.5 | 7901 | 1.964 | 0.852 | 453.5 |
| *Emberiza citrinella* | 13.0 | 5544 | 2.026 | 0.757 | 26.75 |
| *Emberiza schoeniclus* | 11.25 | 36414 | 0.952 | 0.77 | 18.8 |
| *Falco peregrinus* | 17.3 | 1891 | 13.5 | 0.479 | 889.25 |
| *Falco tinnunculus* | 16.4 | 32496 | 14.01 | 0.561 | 174.5 |
| *Ficedula hypoleuca* | 10.9 | 27023 | 14.272 | 0.536 | 14.35 |
| *Fringilla coelebs* | 14.0 | 26945 | 0.787 | 0.593 | 24.2 |
| *Fulica atra* | 20.6 | 54398 | 4.44 | 0.587 | 732.5 |
| *Hirundo rustica* | 11.1 | 39051 | 3.194 | 0.772 | 19.1 |
| *Parus caeruleus* | 9.7 | 79074 | 0.796 | 0.78 | 11.75 |
| *Parus major* | 15.4 | 124485 | 0.797 | 0.722 | 18.5 |
| *Passer domesticus* | 19.7 | 16800 | 0.206 | 0.747 | 30.35 |
| *Phylloscopus trochilus* | 11.7 | 28497 | 2.172 | 0.623 | 9.35 |
| *Pica pica* | 21.7 | 5081 | 1.033 | 0.79 | 228 |
| *Sitta europaea* | 12.9 | 8168 | 1.803 | 0.753 | 23.9 |
| *Strix aluco* | 21.5 | 12174 | 2.12 | 0.63 | 491 |
| *Sturnus vulgaris* | 22.9 | 87505 | 1.1 | 0.762 | 80.5 |
| *Sylvia atricapilla* | 11.6 | 26405 | 17.539 | 0.582 | 18.85 |
| *Turdus merula* | 21.8 | 99685 | 0.264 | 0.597 | 95.85 |
