# Supplementary figures and images for: Dispersal capacity explains the evolution of lifespan variability
Source: Ecol Evol. 2018 Apr 19;8(10):4949–57. doi: 10.1002/ece3.4073 (PMC5980329; doi:10.1002/ece3.4073)

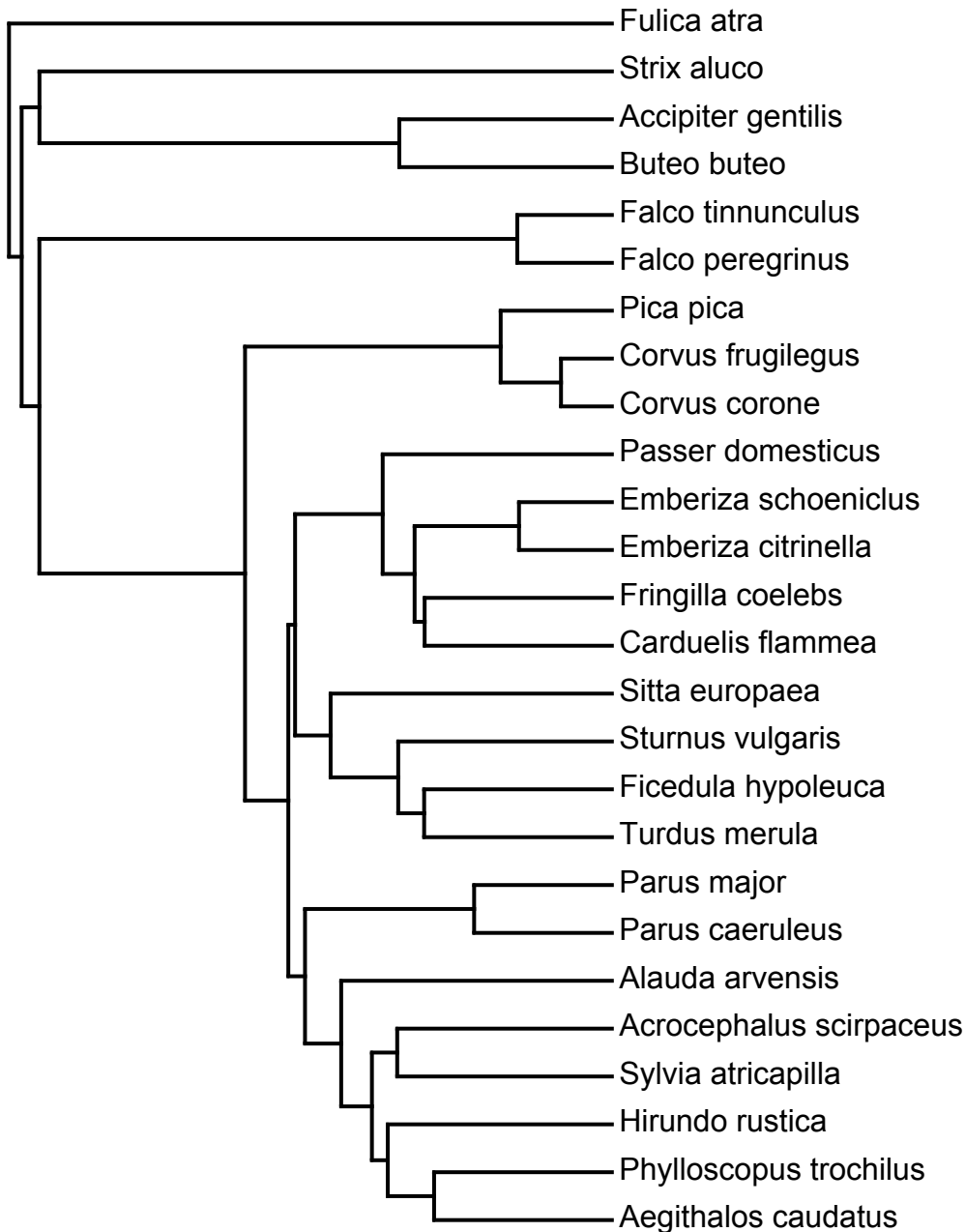

Supplement: Supplementary file 3 [file ECE3-8-4949-s003.pdf]
